# Monitoring *Escherichia coli* in Water through Real-Time Loop-Mediated Isothermal Amplification on Biochips

**DOI:** 10.3390/mi15091112

**Published:** 2024-08-31

**Authors:** Yuxin Wang, Yun-Sheng Chan, Eugene Lee, Donglu Shi, Chen-Yi Lee, Jiajie Diao

**Affiliations:** 1 Department of Cancer Biology, University of Cincinnati College of Medicine, Cincinnati, OH 45267, USA; 2 The Materials Science and Engineering Program, Department of Mechanical and Materials Engineering, College of Engineering and Applied Science, University of Cincinnati, Cincinnati, OH 45221, USA; 3 Advanced Sensing Lab, Digital Futures, University of Cincinnati, Cincinnati, OH 45221, USA; 4 Institute of Electronics, National Yang Ming Chiao Tung University, Hsinchu 30010, Taiwan; eugene@paidge.com; 5 Department of Biomedical Engineering, College of Engineering and Applied Science, University of Cincinnati, Cincinnati, OH 45221, USA

**Keywords:** water quality monitoring, digital microfluidic biochip (DMFB), micro-electrode dot array (MEDA), programmable biochip, loop-mediated isothermal amplification (LAMP), *Escherichia coli*

## Abstract

Access to clean water is fundamental to public health and safety, serving as the cornerstone of well-being in communities. Despite the significant investments of millions of dollars in water testing and treatment processes, the United States continues to grapple with over 7 million waterborne-related cases annually. This persistent challenge underscores the pressing need for the development of a new, efficient, rapid, low-cost, and reliable method for ensuring water quality. The urgency of this endeavor cannot be overstated, as it holds the potential to safeguard countless lives and mitigate the pervasive risks associated with contaminated water sources. In this study, we introduce a biochip LAMP assay tailored for water source monitoring. Our method swiftly detects even extremely low concentrations of *Escherichia coli* (*E. coli*) in water, and 10 copies/μL of *E. coli* aqueous solution could yield positive results within 15 min on a PC-MEDA biochip. This innovation marks a significant departure from the current reliance on lab-dependent methods, which typically necessitate several days for bacterial culture and colony counting. Our multifunctional biochip system not only enables the real-time LAMP testing of crude *E. coli* samples but also holds promise for future modifications to facilitate on-site usage, thereby revolutionizing water quality assessment and ensuring rapid responses to potential contamination events.

## 1. Introduction

Water source monitoring stands as a pivotal and obligatory endeavor crucial for upholding public health and safety standards. At the turn of the 20th century, waterborne diseases like cholera and typhoid posed significant threats to life in the United States [1]. While advancements in providing treated, potable water have notably reduced the incidence of such diseases, waterborne illnesses persist, with an estimated 7.15 million cases occurring annually in the United States alone. These illnesses lead to 601,000 emergency department visits, 118,000 hospitalizations, and 6630 deaths, resulting in approximately USD 3.33 billion in direct health care costs [2]. Regulatory bodies such as the Environmental Protection Agency (EPA) have implemented stringent guidelines mandating the regular monitoring of water sources for harmful pathogens, including *E. coli* [3]. The detection of *E. coli* serves as a critical indicator of fecal contamination and the potential for waterborne diseases, underscoring its vital role in water quality assessment [4]. Compliance with these regulations entails substantial financial investments, with millions of dollars allocated annually to water testing and treatment procedures aimed at safeguarding drinking and recreational water [5,6,7,8]. The development and implementation of innovative testing solutions are imperative to facilitate effective water quality monitoring and management, ultimately bolstering public health and safety efforts.

Traditional methods for detecting *E. coli* in water sources typically involve a labor-intensive process of collecting water samples, culturing bacteria in a laboratory setting, and enumerating the resulting bacterial colonies. While these methods are regarded as reliable, they suffer from significant drawbacks, including prolonged turnaround times, often spanning several days and reliance on specialized laboratory equipment and skilled personnel. These factors hinder their suitability for on-site, rapid testing, thereby delaying responses to potential contamination incidents [9]. Although recent advancements have introduced faster testing methods, such as the quantitative Polymerase Chain Reaction (q-PCR), which offers quicker results compared to traditional culturing techniques, it still necessitates access to laboratory facilities, specialized equipment, and trained personnel, limiting its applicability for immediate on-site testing [10]. Despite technological progress, the reliance on laboratory infrastructure and skilled personnel remains a barrier to achieving immediate on-site testing capabilities. Additionally, the financial implications of bacteriological failures can be substantial, particularly when sample collection locations require transportation via ferry or airplane for re-testing in the laboratory [7]. Recognizing the urgent need for rapid *E. coli* detection to prevent fecal contamination and waterborne diseases, organizations like UNICEF are actively working to define target product profiles for rapid testing methods, emphasizing the importance of ensuring water safety for all [5,11,12]. In light of these challenges, there is a critical demand for new testing methodologies capable of delivering quick, accurate, and easily interpretable results to address the imperative for rapid and reliable *E. coli* detection in drinking water.

Loop-mediated isothermal amplification (LAMP) has emerged as a promising alternative for the rapid and cost-effective detection of *E. coli*. Unlike PCR, LAMP operates at a constant temperature, eliminating the need for thermal cycling and reducing the complexity of required equipment. This simplicity, coupled with LAMP’s sensitivity and specificity, renders it particularly suitable for resource-limited settings and scenarios necessitating swift results [13]. During amplification, the production of P_2_O_7_^4−^ serves as a by-product. The addition of MgSO_4_ to the LAMP buffer facilitates amplification by promoting the reaction between Mg^2+^ and P_2_O_7_^4−^, resulting in the formation of a white precipitate, Mg_2_P_2_O_7_. Consequently, the appearance of this white precipitate serves as a visual indicator of amplification, with turbidity changes in the LAMP sample providing a method for assessing test results [14].

Expanding upon the foundational principles of LAMP, recent advancements have spurred the creation of digital microfluidic biochips, elevating the method’s versatility and efficiency to new heights. The biochips powered by micro-electrode-dot-array (MEDA) technology enable the precise manipulation of minuscule fluid samples while seamlessly integrating multiple functions within a single, compact platform. This innovative approach would be able to streamline the testing procedure and provide real-time monitoring when combined with an imaging system. Moreover, the adaptability and programmability inherent in these biochips offer a distinct advantage over traditional devices, unlocking the potential to conduct a diverse array of biological assays on a unified platform with minimal sample volume requirements [15,16,17]. The transition from traditional culturing techniques to cutting-edge technologies such as LAMP and digital microfluidic biochips marks a substantial progression in the detection of waterborne pathogens. These advancements offer the potential to enhance the efficiency, speed, and accessibility of *E. coli* detection, with the prospect of revolutionizing water quality monitoring practices and securing safer water sources for communities globally.

In this study, we investigated the development and application of a biochip LAMP test specifically designed for water source monitoring. Our focus was on the rapid detection of low concentrations of *E. coli* in water samples, achieved within a 15-minute timeframe using the advanced pattern-control micro-electrode-dot-array (PC-MEDA) biochip technology. This investigation aimed to address the limitations of conventional laboratory-dependent methods and explore the potential for the on-site testing and real-time monitoring of water using a multifunctional biochip system.

## 2. Methods

### 2.1. Materials

We used the 2× WarmStart LAMP Master Mix (WarmStart LAMP Kit (DNA & RNA), #E1700S, New England Biolabs, Ipswich, MA, USA), ethidium bromide stock solution (VWR, Radnor, PA, USA), Molecular Biology-Grade Water (Fisher Scientific, Hampton, NH, USA), and a 1 kb DNA ladder (#N3232S, New England Biolabs).

### 2.2. LAMP Primers

The *E. coli*-specific primers used in this study to target the *malB* gene are in the *E. coli* GenBank sequence (GDB J01648). The *malB* gene is conserved in the *E. coli* lineage and is not shared with other Gram-negative bacteria. Primers were designed based on the study of [18], and the sequences are shown in Table 1. Both 4-primer LAMP and 6-primer LAMP were tested in this study. A 10× 4-primer mix was made with 16 µM FIP, 16 µM BIP, 2 µM F3, and 2 µM B3 in water, while a 10× 6-primer mix contained 16 µM FIP, 16 µM BIP, 2 µM F3, 2 µM B3, 4 µM Loop F, and 4 µM Loop B in water. The concentration of each primer in the 25 µL LAMP reaction mix was 0.2 µM for F3 and B3 primers, 1.6 µM for FIP and BIP primers, and 0.4 µM for Loop F and Loop B.

### 2.3. Sample Collection

Evaluating the specificity and sensitivity of the LAMP reaction involved the use of DH5α competent *E. coli* cultured in Luria–Bertani (LB) broth medium. Live *E. coli* bacterial pellets were obtained by centrifuging the culture at 1200 rpm for 3 min at room temperature using a swing-bucket rotor centrifuge. Subsequently, the pellets were resuspended in nuclease-free water in microcentrifuge tubes and subjected to heat treatment at 95 °C for 10 min. Following heat treatment, the samples were centrifuged at 10,000 rpm for 10 min using a microcentrifuge to collect the supernatant. The collected samples were then frozen at −20 °C for storage before further use.

### 2.4. Dilute Sample Preparation

Tenfold serial dilutions of *E. coli* samples were prepared to test the sensitivity of LAMP detection. The DNA concentration was measured with a NanoDrop 1000 Spectrophotometer (Thermo Fisher Scientific Inc., Waltham, MA, USA). The DNA copy number was calculated through the following equation:

$$DNA\;copy\;number=\frac{c\times 10^{-9}g\times 6.0221\times 10^{23}molecules/mol}{5\times 10^{6}bp\times 650g/mol}$$

where c is the concentration of DNA, 6.0221 × 10^23^ is Avogadro’s constant, 5 × 10^6^ bp is the length of the *E. coli* gene, and 650 is the average mass of 1 bp of DNA.

The expected copy numbers for each *E. coli* sample are shown in Table 2. The tested DNA concentration of the Original *E. coli* sample was measured with a NanoDrop Spectrophotometer as 218.5 ng/μL, and the calculated copy number was 4.04870 × 10^7^ copies/μL. Sample 1 was diluted four times from the Original sample to achieve the expected concentration of 1 × 10^7^ copies/μL. The concentration measurement was performed the same way as the Original sample, and the calculated result of Sample 1 matched the expectation. Tenfold titration was used to prepare Samples 2 to 7. The concentration measurement was performed for Samples 2 and 3, while other samples were too diluted to be measured.

### 2.5. LAMP Assay

A mix of 12.5 µL of 2× LAMP Master Mix, 2.5 µL of 10× primer mix, and 2 µL of the *E. coli* sample was used as the positive sample, whereas the same volume of nuclease-free water was used for the negative control. The testing solution was filled with nuclease-free water until a final volume of 25 µL was obtained.

To commence the experiment, a 1.5 µL volume of the mixed LAMP solution was carefully applied onto the biochip, which was then covered with indium tin oxide (ITO) glass. Silicon oil was used to enclose the droplet on the biochip. The remaining 23.5 µL of the solution was kept in PCR tubes for subsequent processing. In the testing area, the biochip provided an incubation temperature of 65 °C for either 30 or 45 min. Throughout the procedure, a microscope was utilized to observe and monitor the entire process, with the proceedings also being recorded via video. The remaining sample in the PCR tubes was subjected to incubation using a Programmable Thermal Controller PTC-100 (MJ Research Inc., Saint-Bruno-de-Montarville, QC, Canada), with the incubation temperature set at 65 °C for the required duration. To evaluate the end-point results and detect nucleic acid amplification in the tube samples, 1% agarose gel containing 0.5 µg/mL of ethidium bromide (EB) was employed.

## 3. Biochip System

In our investigation, we employed an advanced PC-MEDA biochip, meticulously crafted using standard 0.35 µm 2P4M Complementary Metal Oxide Semiconductor (CMOS) technology. Distinguished by its array of 5400 micro-electrodes (MEs), this biochip is specifically engineered for digital microfluidic operations and precise temperature regulation, surpassing conventional biochips in electrode size, quantity, power efficiency, and the use of high-performance TiN/Al/TiN materials for integration. The PC-MEDA biochip operates on an intricate pattern-control mechanism, employing 2D binary patterns to finely manipulate microfluidic processes and temperature profiles. This functionality is achieved through the principles of electro-wetting-on-dielectric (EWOD), allowing for the manipulation of liquid droplets confined between the biochip and an ITO glass plate [16,19]. Notably, the biochip boasts real-time feedback control facilitated by built-in capacitive sensing circuits, enabling the continuous monitoring of droplet position and size. This dynamic system enables the reconfiguration of electrode patterns to optimize the biochip’s performance for diverse applications, encompassing digital microfluidics, temperature profiling, and capacitive sensing.

**Figure 1:**
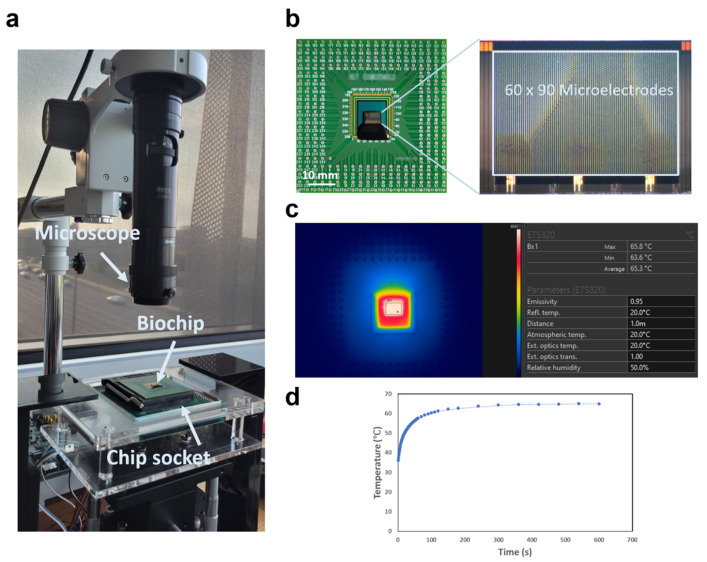
(**a**) The setup of the biochip test platform. (**b**) A photo of the PC-MEDA biochip and a micro-photo of the testing area. (**c**) A thermal image from the infrared camera of the biochip under the heating mode (Bx 1 was selected as the whole testing area). (**d**) Temperature vs. time curve of the biochip from 0 s to 600 s when the LAMP mode was started.

Each micro-electrode within the biochip is individually programmable, affording flexibility in executing intricate bioassays. The biochip’s design features a double-layer structure, with the top layer dedicated to microfluidic operations and the second layer serving dual functions: temperature profiling and acting as a shielding layer. This multifaceted design enables the precise manipulation of bioassay conditions, rendering the PC-MEDA biochip an optimal platform for conducting the LAMP assay. Furthermore, employing the biochip significantly reduces the sample volume required for the LAMP assay, presenting a cost-effective and efficient alternative to conventional methods. Additionally, this pioneering biochip system played a pivotal role in our study, facilitating real-time visualization and monitoring of the LAMP reaction process, which underscores its potential as a potent tool for rapid, sensitive, and portable nucleic acid detection. Figure 1a illustrates both the biochip system and the chip utilized in our experimentation. The main part of the system includes a biochip socket and a microscope. Both are controlled by a computer. Figure 1b is a real product display of the PC-MEDA biochip with a microscopic image of its testing area. An infrared (IR) camera was otherwise employed to measure the LAMP mode temperature during the presetting and adjustment stages, with the heating curve duly recorded. An IR camera image with measured data provided by FLIR software (FLIR Tools 6.4) is shown in Figure 1c as an example of the real conditions during the run of the LAMP heating mode.

## 4. Results and Discussion

### 4.1. E. coli LAMP Assay Feasibility Test

In this study, we employed both 4-primer LAMP and 6-primer LAMP assays to detect *E. coli* in aqueous solution samples using a biochip test platform. Table 1 provides detailed information on the primers utilized in the assays. The key distinction between these two methods lies in the inclusion of two additional loop primers in the 6-primer LAMP assay. These loop primers are known to enhance the efficiency of the amplification reaction, potentially accelerating the presentation of results or increasing sensitivity, particularly in samples with low concentrations of the target organism. To assess the efficacy of both primer mixes for *E. coli* detection, we utilized an Original *E. coli* sample in our experiment. This crude sample underwent a heating process at 95 °C to inactivate the bacteria and expose their DNA copies in the solution, eliminating the need for DNA extraction steps. This simplified sampling method holds promise for field applications where chemical reagents and complex equipment may be impractical.

**Figure 2:**
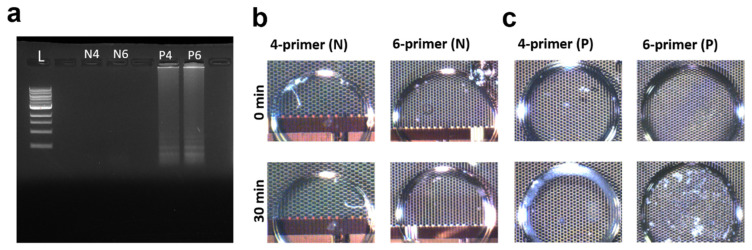
(**a**) Agarose gel results of the *E. coli* LAMP assay. L: 1 kb DNA ladder. N4: negative control of 4-primer LAMP. N6: negative control of 6-primer LAMP. P4: positive sample of 4-primer LAMP. P6: positive sample of 6-primer LAMP. Microscope images of *E. coli* LAMP assay on the biochip at 0 min and 30 min: (**b**) negative control samples and (**c**) positive samples.

Two detection methods were employed: traditional end-point in-tube heating LAMP with agarose gel results (Figure 2a) and real-time biochip LAMP with microscope images (Figure 2b,c). Both methods successfully detected *E. coli* in the Original sample (details of the sample are shown in Table 2).

Figure 2a depicts the agarose gel results, with positive samples exhibiting a distinct ladder-like pattern while negative samples show no signal. Figure 2b shows the microscope images of negative control samples on the biochip; the upper-row images were recorded at 0 min (before reaction), and the lower-row images were from the same sample recorded after 30 min of incubation. The droplets looked clear before and after the reaction, without any visible change in the samples, which indicates that no amplification happened. In contrast, Figure 2c showcases the Original sample on the biochip, where initially transparent droplets developed a white precipitate in the positive samples after 30 min, indicating amplification. The background of the droplets was the testing area on the chip, which was full of micro-electrodes; some droplets touched the edge of the area, making their shape change to partially round. All images contain complete views of the samples.

These findings demonstrate the accuracy of both the 4-primer and 6-primer LAMP assays for *E. coli* detection and highlight the feasibility of conducting this detection process on a biochip test platform.

### 4.2. Detectable Concentration Limit Study

**Figure 3:**
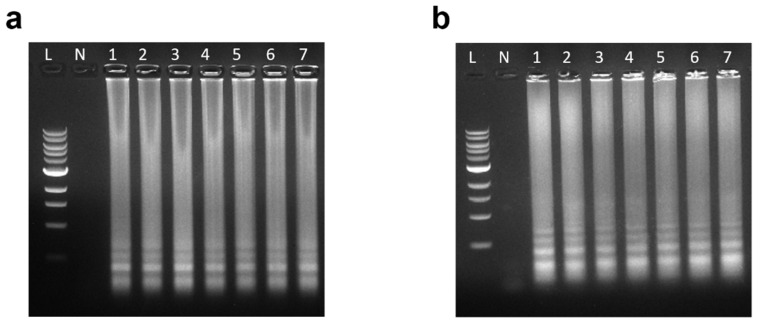
Agarose gel results of the sensitivity test for the LAMP assay. The lanes labeled 1–7 correspond to Sample 1–Sample 7 in Table 1. L: 1 kb DNA Ladder. N: negative control. (**a**) Four-primer LAMP; (**b**) six-primer LAMP.

Subsequently, a series of diluted *E. coli* samples were prepared in accordance with the methods outlined. As detailed in Table 1, these samples ranged from a relatively high concentration (1 × 10^7^ copies/μL) to a low concentration (10 copies/μL) and were designated Sample 1 through Sample 7. Each *E. coli* sample, along with negative control samples (pure water), was individually mixed with both the 4-primer LAMP solution and the 6-primer LAMP solution. The sensitivity of the LAMP assay for *E. coli* detection was assessed using in-tube heating and agarose gel analysis, with the results presented in Figure 3. Remarkably, both the 4-primer and 6-primer LAMP assays yielded identical outcomes: all samples, from Sample 1 to Sample 7, exhibited a distinct ladder-like pattern, while the negative control showed no signal. In summary, the LAMP reaction successfully detected *E. coli* at concentrations as low as 10 copies/µL. Furthermore, the sensitivities of both the 4-primer and 6-primer LAMP assays proved to be comparable, indicating their suitability for detecting low concentrations of *E. coli* in samples.

### 4.3. Time Effect on Amplification for Low-Concentration Sample

In the conventional testing method, LAMP samples are enclosed in tubes with lids and heated in a heating block. Post-reaction, the sample must be extracted from the tube for gel electrophoresis, posing a risk of contamination due to the high concentration of gene copies post-exponential amplification. Additionally, the preparation of agarose gels, loading buffer, and electrophoresis instruments is required. In contrast, our biochip system offers a one-step testing solution with real-time imaging capabilities. Upon loading the samples, initiating the LAMP mode triggers rapid temperature attainment by the electrode array on the chip, allowing the entire reaction process to be observed and recorded via the microscope camera in real time. This facilitates the visual identification of positive samples without the need for specialized equipment. To assess the performance of the real-time biochip system in detecting low-concentration *E. coli*, a time-related test was devised. Sample 7 (10 copies/μL) served as the positive sample, with real-time microscope images captured every 5 min. Simultaneously, a series of in-tube heating-positive samples were prepared, each heated for 0 to 40 min, corresponding to the time points of the recorded images.

**Figure 4:**
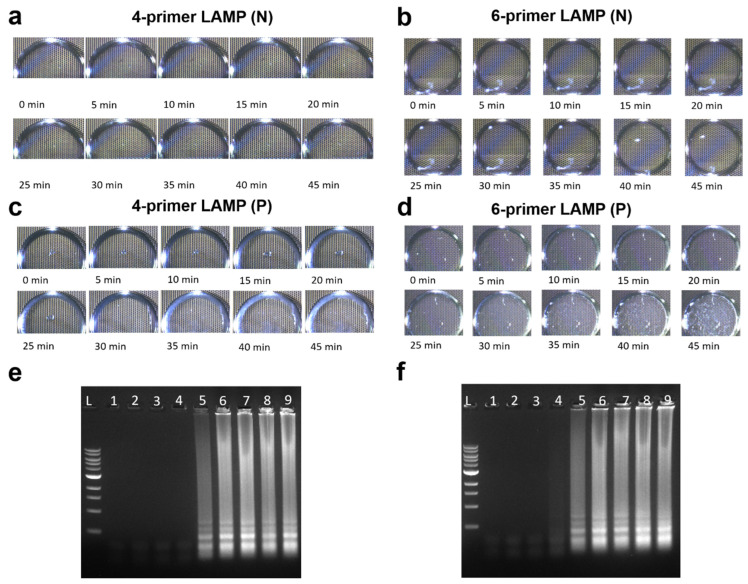
Real-time images of samples on the biochip during the LAMP assay. (**a**) A negative sample with 4-primer LAMP; (**b**) a negative sample with 6-primer LAMP; (**c**) Sample 7 with 4-primer LAMP; and (**d**) Sample 7 with 6-primer LAMP. (**e**,**f**) Agarose gel results for Sample 7 with 4-primer LAMP and 6-primer LAMP. The lanes labeled 1–9 correspond to the time at 0 min, 5 min, 10 min, 15 min, 20 min, 25 min, 30 min, 35 min, and 40 min. L: 1 kb DNA ladder.

Figure 4a–d depict a series of real-time tracking images during the LAMP test on the biochip. Figure 4a,b show negative samples with 4-primer and 6-primer LAMP, respectively, remaining transparent throughout the 45-minute duration, which is akin to the negative samples in Figure 2. Figure 4c,d illustrate positive samples with 4-primer and 6-primer LAMP, respectively, both exhibiting similar changes. Initially transparent, a faint white cloud appeared in the 4-primer sample at 15 min, while in the 6-primer sample, it appeared at 10 min, gradually intensifying before stabilizing after 35 min. Gel electrophoresis was conducted for the in-tube samples, depicted in Figure 4e (left: 4-primer) and 4f (right: 6-primer). For the 4-primer samples, the first positive signal emerged at 20 min, strengthening by 25 min before plateauing. In contrast, the 6-primer samples showed a weaker positive signal at 15 min, strengthening at 20 and 25 min before stabilizing. The results, as depicted by both images and gels, affirm the accuracy of the LAMP assay, with positive signals observed on the biochip 5 min earlier than on the gel. Moreover, the 6-primer assay demonstrated slightly higher efficiency, yielding earlier or stronger signals in the same early stage compared to the 4-primer assay. Ultimately, while the 4-primer LAMP remains a cost-effective choice, the 6-primer LAMP offers enhanced efficiency.

### 4.4. Future Direction

The application of real-time LAMP on biochips has proven to be an ideal solution for water source monitoring tasks. Collecting and preparing water samples has become remarkably straightforward, requiring just a 10-minute heating step. Gone are the days of reliance on expensive equipment and chemicals; now, a small heating block or even a standard kettle suffices for the heating process. With the biochip system alone, the entire testing process is streamlined, eliminating the need for multiple devices such as an incubator, gel electrophoresis device, and gel reader, as required by traditional LAMP. Moreover, the real-time visibility of the reaction replaces the incubation, reduces the gel steps to one, and reduces the time from over 1 hour to just 15 min. Our focus is on enhancing the portability of the new-generation biochip system. A smartphone app now serves as the system controller, replacing the need for a computer, while a 65-watt USB-C power adapter provides the necessary power supply. Ongoing efforts are directed toward refining biochips to better accommodate the LAMP assay. Sample pretreatment will be conducted directly on the chips, leveraging the controlled heating capability to swiftly reach 95 °C and cool down rapidly upon current blockage. Subsequently, the premixed LAMP solution will be applied to the biochip, with micro-electric currents facilitating the mixing of water samples and the LAMP solution based on EWOD principles.

The final step involves heating and observing the amplification reaction, as previously demonstrated. Furthermore, the versatility of the LAMP assay extends beyond *E. coli* detection; by mixing primers targeting other viruses or bacteria, a universal assessment of water contamination can be achieved. Alternatively, individual primer sets can be utilized to determine the presence or absence of specific species. This real-time biochip LAMP technology heralds a significant advancement in on-site water source monitoring, offering a streamlined, one-step solution.

## 5. Conclusions

In summary, this study represents a leap forward in the detection of *E. coli* in water sources, transcending traditional methods through the utilization of LAMP and digital microfluidic biochips. By adopting these advanced technologies, we have demonstrated the capability for the rapid, sensitive, and cost-effective detection of *E. coli*, which is crucial for monitoring water quality and ensuring public health. While conventional methods remain dependable, their time-intensive nature and reliance on specialized laboratory environments limit their suitability for on-site and rapid response scenarios. In contrast, LAMP offers a rapid and accessible alternative, especially when coupled with digital microfluidic biochips, which enable the precise manipulation of minute crude samples and the real-time monitoring of the amplification process. The outcomes of this study underscore the transformative potential of these technologies in revolutionizing water quality monitoring practices. They hold the promise of swiftly and accurately assessing the safety of drinking water and recreational water, thereby shielding communities from the consequences of waterborne diseases.

## Figures and Tables

**Table 1:** Information about *E. coli* primers.

Primer	Name	Sequence (5′–3′)
Outer	F3	5′-GCCATCTCCTGATGACGC-3′
	B3	5′-ATTTACCGCAGCCAGACG-3′
Inner	FIP	5′-CTGGGGCGAGGTCGTGGTAT-TCCGACAAACACCACGAATT-3′
	BIP	5′-CATTTTGCAGCTGTACGCTCGC-AGCCCATCATGAATGTTGCT-3′
Loop	Loop F	CTTTGTAACAACCTGTCATCGACA
	Loop B	ATCAATCTCGATATCCATGAAGGTG

**Table 2:** Concentration and number of DNA copies for the **Original** *E. coli* samples in water and dilution: **Sample 1**–**Sample 7**. N/A: no relevant information.

Samples	Expected Number of DNA Copies/μL	DNA Concentration (ng/μL)	Calculated Number of DNA Copies/μL	Dilution Ratio (Dilute from the Last Sample)
Original	N/A	218.5	4.04870 × 10^7^	N/A
1	1 × 10^7^	56.26666667	1.04260 × 10^7^	1:4
2	1 × 10^6^	5.666666667	1.05001 × 10^6^	1:10
3	1 × 10^5^	0.566666667	1.05001 × 10^5^	1:10
4	1 × 10^4^	N/A	N/A	1:10
5	1 × 10^3^	N/A	N/A	1:10
6	1 × 10^2^	N/A	N/A	1:10
7	1 × 10^1^	N/A	N/A	1:10

## Data Availability

The original contributions presented in the study are included in the article, further inquiries can be directed to the corresponding authors.
